# Reverse zoonosis of coronavirus disease-19: Present status and the control by one health approach

**DOI:** 10.14202/vetworld.2021.2817-2826

**Published:** 2021-10-30

**Authors:** R. Kumar Pramod, Asha V. Nair, Padmakar Kamalakar Tambare, Kanchana Chauhan, T. Vinay Kumar, R. Anju Rajan, Blessy M. Mani, Muhasin Asaf, Amit Kumar Pandey

**Affiliations:** 1 Small Animal Facility, Translational Health Science and Technology Institute, NCR Biotech Science Cluster, Faridabad, Haryana, India; 2 Department of Chemistry, Indian Institute of Technology Kharagpur, Kharagpur, West Bengal, India; 3 Krishi Vigyan Kendra, Kottarakkara, Kollam, Kerala, India; 4 Inter University Centre for Biomedical Research and Super Speciality Hospital, Kottayam, Kerala, India; 5 Department of Animal Breeding and Genetics, KVASU, Wayanad, Kerala, India; 6 Mycobacterial Pathogenesis Laboratory, Translational Health Science and Technology Institute, NCR Biotech Science Cluster, Faridabad, Haryana, India.

**Keywords:** mink, non-human primate, one health, reverse zoonosis, severe acute respiratory syndrome coronavirus 2

## Abstract

The recent coronavirus disease (COVID-19) outbreak is one of its kind in the history of public health that has created a major global threat. The causative agent, severe acute respiratory syndrome coronavirus 2 (SARS-CoV-2) has a zoonotic source and hence, reverse zoonosis (disease transmission from humans to animals) increases the risk and rate of SARS-CoV-2 infection. Serological and molecular analyses and experimental infection studies have identified SARS-CoV-2 infection in several animal species in various countries. Different domestic and wild animals, including cats, dogs, tigers, lions, puma, snow leopard, minks, and pet ferrets, are infected naturally with SARS-CoV-2, mostly through suspected human to animal transmission. In addition, in vivo experimental inoculation studies have reported the susceptibility of cats, ferrets, hamsters, Egyptian fruit bats, and non-human primates to the virus. These experimentally infected species are found to be capable of virus transmission to co-housed animals of the same species. However, SARS-CoV-2 showed poor replication in livestock species such as pigs, chickens, and ducks with no detection of viral RNA after the animals were deliberately inoculated with the virus or exposed to the infected animals. As the pets/companion animals are more susceptible to COVID-19, the infection in animals needs an in-depth and careful study to avoid any future transmissions. The one health approach is the best inter-disciplinary method to understand the consequences of viral spread and prevention in novel host populations for the betterment of public health. Further in this review, we will explain in detail the different natural and experimentally induced cases of human to animal SARS-CoV-2 infection.

## Introduction

The pandemic of novel zoonotic coronavirus disease 2019 (COVID-19) caused by the severe acute respiratory syndrome coronavirus 2 (SARS-CoV-2) was first reported in Wuhan, China [1,2], which is rapidly infecting the global population with over 182 million confirmed cases and more than 3,954,324 deaths as of June 30, 2021 [3]. Within the past two decades, SARS-CoV-2 is the third coronavirus that has challenged the human race; the first and second being the SARS-CoV and the Middle East respiratory syndrome coronavirus (MERS-CoV), emerged during 2002 and 2012, respectively [4]. Among the three coronaviruses, SARS-CoV-2 is responsible for the highest number of infections and fatality causing a significant global economic loss. Despite extensive research, we are struggling to combat the public health concerns raised by viral infections, high viral genome mutations, the development of sensitive diagnostic kits, effective and safe therapeutics and vaccine.

The origin of SARS-CoV-2 is still controversial. It had been thought to have originated in animals that later on got transmitted to the human population. The isolation of genetically related coronaviruses from Rhinolophus bats [5,6] and Malayan pangolins (*Manis javanica*) [7-9] has led to the hypothesis that the zoonotic origin of the SARS-CoV-2 is most likely from one of these species. Furthermore, the genome analysis of SARS-CoV-2 showed 96.2% identity with the bat RaTG13 coronavirus genome [6]; however, the Spike protein of RaTG13 coronavirus of the bat *Rhinolophus affinis* did not specifically bind to the human angiotensin-converting enzyme 2 (ACE2) receptor due to its significant difference in the receptor-binding domain [10]. On the contrary, the high receptor homology of ACE2 of humans, cats, ferrets, etc., to the Spike protein of SARS-CoV-2 helped in effective binding and successful transmission across both these animals and humans [11]. SARS-CoV-related coronaviruses were also observed in masked-palm civets (*Paguma larvata*) and raccoon dogs (*Nyctereutes procyonoides*) [12,13], showing the wide pool of mammalian species infected by SARS-CoV and SARS-CoV-2 related viruses suggesting interspecies transmission of these viruses. However, the definite source and route of SARS-CoV-2 ingress into the human race are yet to be established. SARS-CoV-2 infection in animals has been reported from several countries, both in domestic and zoo animals, namely, cats, dogs, farmed minks, tigers, and lions [14]. Evidence shows that the infection is due to close contact with people infected with COVID-19 [15,16]. Further, laboratory studies in cats, ferrets, and hamsters through experimental infection with SARS-CoV-2 demonstrated the ability of these animals of the species to infect one another if co-housed [17,18]. Also genetic changes, the virus gains randomly during its replication cycle can make it endemic in some animal populations, including domestic pets. Studies have also been conducted in non-human primate models to understand and identify the key features of SARS-CoV-2 infection [19,20]. However, experimental studies show poor replication of SARS-CoV-2 in livestock, namely, pigs, chickens, and ducks, where no viral RNA was observed in the animals even after being deliberately inoculated with the virus or in those exposed to the infected animals [21,22].

**Figure-1:**
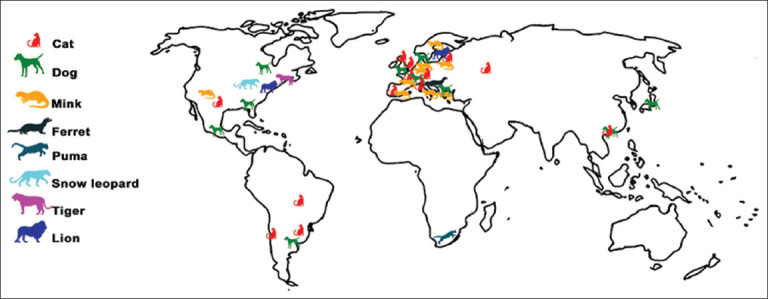
World map shows locations of severe acute respiratory syndrome coronavirus 2 (SARS-COV-2) infected animals. SARS-CoV-2 infection in animals were reported from countries worldwide, including Belgium, France, USA, Italy, Spain, Netherlands, Denmark, Hong Kong, Germany, Argentina, England, Brazil, Russia, Lithuania, South Africa, Switzerland, Sweden, Japan, Greece, Chile, Mexico, Slovenia, Estonia, Bosnia and Herzegovina, Poland and Latvia. SARS-COV-2 infection was more reported in animals from USA and European countries. Cats, dogs and minks are the most affected animals [Source: Map was prepared by the corresponding author].

**Figure-2:**
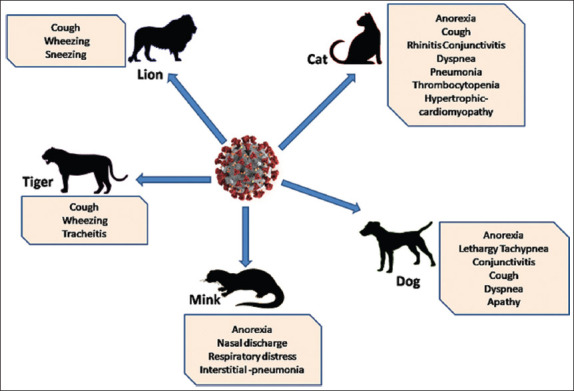
Clinical signs and symptoms observed in some of the severe acute respiratory syndrome coronavirus 2 infected animal species. The infected animals mainly showed respiratory symptoms [Source: Figure was prepared by the corresponding author].

The very first case of SARS-CoV-2 transmission from human to animal was reported in Hong Kong, where a 17-years-old Pomeranian dog was infected [23]. Whereas, the first case of asymptomatic infection in a companion animal was in a cat from the USA [24]. Thenceforth several cases of SARS-CoV-2 infection in animals were reported from more countries, namely Belgium, France, USA, Italy, Spain, Netherlands, Denmark, Hong Kong, Germany, Argentina, England, Brazil, Russia, Lithuania, South Africa, Switzerland, Sweden, Japan, Greece, Chile, Mexico, Slovenia, Estonia, Bosnia and Herzegovina, Poland, and Latvia (Figures-1 and 2). According to the World Organization for Animal Health, erstwhile the Office International des Epizooties (OIE), any confirmed SARS-CoV-2 infection in an animal should be reported to the OIE as per the Terrestrial Animal Health Code [14]. Further in this review, we will explain in detail the different natural and experimentally induced cases of human to animal SARS-CoV-2 infection.

## Natural Infection

### Cats

The first case of SARS-CoV-2 transmission from human to cat was reported from Belgium on 6^th^ March 2020 after the owner was diagnosed with COVID-19 [14,25]. This was the third reported case of human to animal transmission, the first two cases being from dogs in Hong Kong. SARS-CoV-2 RNA was detected in the cat’s nasopharyngeal swab samples and vomitus as well as feces. The immunoglobulin specific for the virus was also found in convalescent-phase serum samples. Later on, in France, real-time polymerase chain reaction analysis of the rectal swab of a cat tested positive for the virus; which was the first reported case of natural infection of a cat in France, probably from their infected owners [16] and the Cat SARS-CoV-2 belonged to the phylogenetic clade A2a, same as most of the French human SARS-CoV-2 [16]. The cat SARS-CoV-2 sequence showed eight nucleotide mutations in comparison to the Wuhan reference sequence based on the 94% covered genome. Furthermore, out of the five amino acid mutations, four were in ORF1ab (T265I, I3512T, K4377E, and P4715L). Further, the cat SARS-CoV-2 genome presented the amino acid mutation, D614G, in its spike glycoprotein, specific to the clade A2 enclosing most of the French SARS-CoV-2 sequences [16]. The Institute of Agrifood Research and Technology reported the first case of cat SARS-CoV-2 infection in Spain on May 8, 2020, in a 4-year-old cat from a household with a confirmed case of human COVID-19 [26]. The animal showed severe respiratory distress and was diagnosed with hypertrophic cardiomyopathy. Later on, the oropharyngeal swab of an asymptomatic 8-year-old female domestic European cat was also tested positive for SARS-CoV-2 in Spain [27].

During the first wave of the COVID-19 pandemic, the human-to-cat transmission of SARS-CoV-2 was reported from the UK with mild or severe respiratory diseases. The oropharyngeal swab of a cat with rhinitis and conjunctivitis tested positive for SARS-CoV-2 infection [28]. Afterward, another cat with pathological changes in lungs accordant with viral pneumonia tested positive for COVID-19. High throughput sequencing of the SARS-CoV-2 from the first cat revealed five single nucleotide polymorphisms in the feline viral genome compared to the nearest UK human SARS-CoV-2 sequence. A comparison with nine other feline-derived SARS-CoV-2 sequences from other parts of the world revealed no shared cat-specific mutations [28]. In Hong Kong, a 7-year-old female domestic shorthair cat showed SARS-CoV-2 nucleoprotein gene copy number of log 10 6.3/mL, log10 5.6/mL, and 3.2 log10/mL in nasal, oral, and rectal swabs, respectively [29]. The genome analysis of the cat showed a high sequence identity (99.8%) to the owner.

Twenty-five cats had been reported with SARS-CoV-2 infection in the USA till December 11, 2020 (https://www.aphis.usda.gov/aphis/ourfocus/animalhealth/sa_one_health/sars-cov-2-animals-us). The virus showed its presence in two pet cats in New York, marking the first confirmed case in companion animals in the United States. On May 13, 2020, the first cat SARS-CoV-2 infection in Germany was confirmed by the Bavarian Office for Health and Food Safety, Erlangen, Bavaria [14]. Thereafter, one more cat tested positive for SARS-CoV-2 infection in Germany. In September, Argentina carried out a research project among the pets living with COVID 19 infected people; interestingly two out of four cats were tested positive for SARS-CoV-2 [14]. There on the Federal University of Paraná, in Brazil, diagnosed the SARS-CoV-2 virus in oral and nasal swabs collected from an asymptomatic female domestic cat [14]. On February 1, 2021, a cat was diagnosed with SARS-CoV-2 infection in Latvia [14].

### Dogs

The first case of human to animal SARS-CoV-2 transmission in the whole world was reported when 15 dogs from households with known COVID-19 cases were quarantined and tested in Hong Kong from February 2020 to March 2020. A 17-year-old male Pomeranian and 2.5-year-old male German shepherd dogs were tested positive for SARS-CoV-2 [23]. However, both the animals did not show any specific clinical signs. Genome analysis demonstrated that viral genetic sequences from these two dogs were identical to the virus detected in the respective human cases. The first confirmed case of SARS-CoV-2 in a dog (German Shepherd) in the USA was reported on June 2, 2020, which got infected from its owners [14]. Further, SARS-CoV-2 specific antibodies were found in a second dog in the household, indicating the presence of virus, although the dog did not develop clinical signs of infection. In Argentina, four dogs were infected by COVID-19 and among these, one was affected by conjunctivitis, cough, dyspnea, and weakening, but rests were asymptomatic [14]. The National Institute for Infectious Diseases reported the first case of SARS-CoV-2 in a Japanese dog by RT-PCR and sequencing analysis on July 30 and August 3, 2020, respectively. Later on, one more dog was found infected in Japan [14]. The Friedrich Loeffler Institute of Germany confirmed two cases of SARS-CoV-2 in dogs [14]. Subsequently, the Ministry of Agriculture, Mexico reported COVID-19 infection in five dogs [14]. Further, Bosnia and Herzegovina also reported a SARS-CoV-2 positive case [14].

### Minks and ferrets

The infection by SARS-CoV-2 in farmed mink (*Neovison vison*) resulted in increased mortality creating a great economic loss to mink farming. The countries like Netherlands, Denmark, the USA, France, Spain, Italy, Sweden, Canada, Lithuania, and Greece have reported mink infections to the World Organization for Animal Health [14]. The first reported cases were from two farms in Netherlands in April 2020. An in-depth investigation was conducted after the initial identification of SARS-CoV-2 infection to understand the potential transmission routes and to perform an environmental and occupational risk assessment [15]. About 68% of the tested farmworkers, relatives, or contacts were infected with SARS-CoV-2, indicating the risk of transmissibility to humans from SARS-CoV-2 infected mink. When compared to Wuhan reference sequence NC_045512.2, several non-synonymous mutations were identified among the mink sequence genome but the analyzed sequences did not display any particular amino acid variations. Oude Munnink *et al*. [15] reported that the genome sequence of SARS-COV-2 from the local Netherland population reflected the general diversity of SARS-CoV-2 seen in the Netherlands and were not related to the clusters of mink sequences in the mink farms. This result indicated the absence of spill-over from minks to the people living near mink farms in the Netherlands.

Danish authorities reported an extensive spread of the SARS-CoV-2 virus across 207 mink farms in Denmark from June to December 2020 [14] and researchers have identified 170 coronavirus variants in the viral samples from 40 mink farms. Denmark’s Statens Serum Institute identified seven unique mutations in the spike protein of SARS-CoV-2 variants co-circulating in mink and humans (https://www.who.int/csr/don/03-december-2020-mink-associated-sars-cov2-denmark/en/). On November 4, 2020, Denmark finally decided to cull all the minks raised on the farm as it became impossible to prevent the spread of infection from one farm to another, or from animals to humans, as the mink population acted as a reservoir that contributed to the ongoing transmission in Denmark. According to the Greek agriculture ministry, two mink farms in Northern Greece were also affected with COVID-19 infection and the viral strain found there was identical to that of the humans [14,30]. Recently, four minks were affected with SARS-CoV-2 infection in Poland [14]. Further, the city of Celje, Slovenia reported a COVID-19 case in a pet ferret living in a COVID-19 positive household [14].

### Wild animals

In the 1^st^ week of April 2020, the United States Department of Agriculture’s National Veterinary Services Laboratories (NVSL) confirmed SARS-CoV-2 infection in a female Malayan Tiger at the Bronx zoo, New York [14,31]. This was the first confirmed animal SARS-CoV-2 infection in the USA and the first to be reported in a non-domestic species in the world. In continuation, the zoo tested all their tigers and lions for COVID-19 and a total of four tigers and three lions were diagnosed with SARS-CoV-2 infection. The SARS-CoV-2 genome sequence from the first tiger was identical to the viral sequence from a tiger keeper and to the other human SARS-CoV-2 strains detected in New York (NY-CDC-2929 [MT304486] and NY-QDX-00000001 [MT452574.1]) [31]. The similarity in the viral genomes and the absence of new animal introductions to the area, support the conclusion of the transmission of the virus from an infected keeper(s) to the tigers. One snow leopard at a zoo in Kentucky, USA was confirmed positive for SARS-CoV-2 at the NVSL based on molecular testing (RRT-PCR and sequencing). The affected snow leopard exhibited mild respiratory clinical illness but recovered from it [14]. On December 11, 2020, the Center for Epidemiology and Animal Health, USA, reported SARS-CoV-2 infection in a free-ranging, wild American Mink from Utah [14].

In the 1^st^ week of December 2020, Spain veterinary authority confirmed SARS-CoV-2 infection among four lions in the Barcelona zoo [14]. Two staff at the zoo were also tested positive for COVID-19. Later, a zoo puma from Johannesburg, South Africa, also tested positive for SARS-COV-2 after coming in contact with an infected animal handler [14]. On January 14, 2021, the laboratory SYNLAB Eesti OÜ confirmed SARS-CoV-2 in a 17-year-old male zoo lion in Tallinn, Estonia [14]. Recently, Chandler *et al*. [32] demonstrated the presence of SARS-CoV-2 antibodies in the wild deer from four states (Michigan, Pennsylvania, Illinois, and New York) of USA.

## Experimental Infection

The establishment of suitable animal models of SARS-CoV-2 infection is essential to understand the pathogenicity of the infection, the development of effective anti-viral drugs and vaccines. Experimental infection and transmission studies also help in the identification of potential intermediate hosts in the evolution of the virus from its natural animal reservoir. Research reports indicate that cats, ferrets, hamsters, and non-human primates can be infected with SARS-CoV-2 and can transmit the virus to naïve animals under experimental conditions.

### Carnivores, bats, shrews, and deer

The transient infection of SARS-CoV-2 in Egyptian fruit bat (*Rousettus aegyptiacus*) was reported by intranasal inoculation [21]. The virus was detected in the nasal cavity, trachea, lung, and lung-associated lymphatic tissue similar to the characteristics of a reservoir host. During the period of study, one of the three bats which were in contact with the infected bat also contracted infection with the virus. However, in the case of ferrets, intranasal inoculation showed more efficient virus replication and transmission to all direct contact animals, but with no clinical signs [21]. Mild rhinitis was associated with viral antigen detection in the respiratory and olfactory epithelium of the infected ferrets, suggesting the efficient spread of SARS-CoV-2 in ferrets similar to subclinical human infection. Subsequently, a study was conducted to evaluate nasal shedding of the SARS-CoV-2 virus from inoculated cats to the non-infected ones [17]. This research identified the presence of the virus in all three inoculated cats by day 3 and in the cats that were co-housed with them. Gaudreault *et al*. [33] conducted an in-depth analysis of SARS-CoV-2 infection in cats and identified tracheobronchoadenitis of submucosal glands with the presence of viral RNA in the airways of the viral inoculated cats. Antibodies against SARS-CoV-2 were produced in principal and sentinel animals. Transmission of SARS-CoV-2 among domestic cats suggests the significance of public health awareness and the relevance to explore the potential chain of human-cat-human transmission.

Richard *et al*. [34] provided the first evidence of efficient air transmission of SARS-CoV-2 between ferrets. They observed a similar pattern of virus shedding in direct contact and indirect recipient ferrets with the inoculated ferrets. Thus, the ferret transmission model is a useful tool to understand transmission dynamics and the molecular basis of the transmissibility of SARS-CoV-2 and other betacoronaviruses. Another study demonstrated the presence of SARS-CoV-2 in the upper respiratory tract of ferrets for up to 8 days, without causing severe disease or death [35]. SARS-CoV-2 was detected in the nasal turbinates, soft palate, and tonsils of four inoculated ferrets. In continuation, the same researchers showed the presence of SARS-CoV-2 RNA in five of the virus inoculated 8-month-old (subadult) outbred domestic as well as in the exposed cats in a cage adjacent to the infected cats. Antibodies against SARS-CoV-2 were detected in all the cats hence demonstrated the efficient replication of SARS-CoV-2 and its transmission through respiratory droplets in cats. In case of dogs, SARS-CoV-2 RNA was found in the rectal swab of virus-inoculated ones; however, no infectious virus was observed in swabs collected from any other orifices in these animals [35]. Although antibodies were detected against SARS-CoV-2 in two dogs inoculated with the virus, another two virus-inoculated dogs and two contact dogs were all seronegative, indicating the less susceptibility of dogs to SARS-CoV-2.

By experimental infection, Zhao and co-workers reported the less susceptible nature of tree shrews (*Tupaiabelangeris*) to SARS-CoV-2 [36]. There were no clinical signs in SARS-CoV-2 inoculated tree shrews during the experiment except for elevated body temperature, especially in female animals. All animals in different age groups showed a low level of virus shedding and replication in tissues and young animals exhibited the highest viral shedding during the early stage of infection. Recently, a group of researchers reported that intranasal inoculation of deer fawns with SARS-CoV-2 causes subclinical viral infection and the shedding of infectious virus in nasal secretions. Moreover, the non-inoculated contact deer got infection from the inoculated animals [37].

### Hamsters

Syrian hamsters showed more susceptibility to SARS-CoV-2 infection with moderate interstitial pneumonia leading to transient mild to moderate disease conditions with the presence of SARS-CoV-2 RNA in the respiratory tract of virus-challenged and naive contact Syrian hamsters [38]. These two groups showed similar histopathological changes and viral N protein expression, confirming the transmission of SARS-CoV-2 to naive contact animals from the inoculated hamsters. Another study reported that the SARS-CoV-2 induced pathological lesions in the lungs of Syrian hamsters are similar to the commonly reported features of COVID-19 patients with pneumonia [39]. Further, it was noticed that SARS-CoV-2 infected hamsters developed neutralizing antibody responses and hence were protected against rechallenge with SARS-CoV-2. Moreover, virus replication in the lung of naïve hamsters was inhibited by the passive transfer of convalescent serum to those animals.

Efficient viral replication in the lower respiratory system and apparent weight loss were observed in inoculated and naturally infected hamsters [18]. The researchers noticed the transmission of the virus from inoculated hamsters to naive in-contact hamsters [18]. Furthermore, Rosenke and co-workers refined viral and host parameters in their hamster model studies and determined the infection-causing dose of SARS-CoV-2 in 50% of animals (ID50) following an intranasal infection method [40]. This indicated the highly susceptible nature of these animals to the infection and also showed that neither the age nor sex of the hamster had an impact on the severity of the disease or its course of infection. The knockout of interleukin 2 receptor gamma prolonged the viral persistence in hamsters exposing the susceptibility of SARS-CoV-2 to adaptive immune control [40]. In Chinese hamsters, high viral replication in the upper and lower respiratory tract accompanied by bronchitis and pneumonia as well as significant body weight loss were observed following viral inoculation suggesting the vulnerability of this species of hamster to SARS-CoV-2 infection [41].

### Non-human primates

Among the non-human primates, cynomolgus macaque, rhesus macaque, green monkeys, and common marmoset were used to assess the pathogenic potential of SARS-CoV-2. Cynomolgus macaque was used to compare the effect of inoculation of SARS-CoV-2 with MERS-CoV and SARS-CoV [19]. Inoculation of SARS-CoV-2 resulted in a productive infection but with the absence of overt clinical signs. The asymptomatic cynomolgus macaque model showed a peak of SARS-CoV-2 shedding in the early course of infection, similar to symptomatic human patients [42]. Moreover, on the 4^th^-day post-inoculation, the SARS-CoV-2 antigen was detected in the ciliated epithelial cells of nasal mucosa, which was not seen for MERS-CoV infection [19] or SARS-CoV [43]. Chandrashekar *et al*. [44] demonstrated SARS-CoV-2 infection-induced protective immunity against re-exposure in rhesus macaques. In another study, rhesus macaques showed respiratory disease for 8-16 days after inoculation with SARS-CoV-2 [45]. This suggested that the rhesus-macaque model recapitulates SARS-CoV-2 infection in humans concerning virus replication and shedding, the presence of pulmonary infiltrates histological lesions and seroconversion. Further, another study demonstrated the presence of SARS-CoV-2 RNA in the respiratory tract of rhesus macaque after challenging with the virus [20]. It was also identified that neutralizing antibodies generated from the primary infection could protect the rhesus macaques from any second challenge by SARS-CoV-2. Old-aged rhesus macaques suffered more severe interstitial pneumonia than young macaques after the intratracheal inoculation with SARS-CoV-2 [46].

Another animal model used to study the SARS-CoV-2 challenge was the African green monkeys [47]. African green monkeys exhibited a high degree of viral replication followed by severe pulmonary lesions, the major characteristic of the disease in human patients. Pulmonary consolidation with hemorrhage that varied in severity between animals and lung lobes was noticed in all the animals. Furthermore, interleukins and other pro-inflammatory cytokines and chemokines were elevated in the serum, similar to humans. Moreover, one of the prominent findings in SARS-CoV-2 infection of humans, a rise in the fibrinogen, was observed in the majority of monkeys. Thus, the researchers concluded that virus replication and respiratory disease can be produced in African green monkeys using a much lower and more natural dose of SARS-CoV-2 than what has been used in other non-human primate experiments. Lu *et al*. [48] suggested that rhesus macaque is a better animal model for COVID-19 than cynomolgus macaque and the common marmoset (*Callithrix jacchus*) through comparison studies. Although the viral genome was observed in the swab and blood samples from these three species, viral load was not detected in the pulmonary tissues of the common marmoset. An increased inflammatory cytokine expression and pathological changes in the pulmonary tissues were observed in rhesus macaque than the other two species showing the varying susceptibilities of old world and new world monkeys to SARS-CoV-2.

### Livestock and poultry

Cattle showed less susceptibility to SARS-CoV-2 infection under experimental conditions [22]. Out of the six cattle inoculated, two were positive for SARS-CoV-2. Viral RNA was detectable only shortly after inoculation and by day 2 of inoculation the animals became negative for SARS-CoV-2 and there was no intraspecies transmission observed in the contact cattle [22]. In another study, SARS-CoV-2 challenged pigs and chickens showed no signs of infection, and all the swabs, organ samples, and contact animals, remained negative for SARS-CoV-2 RNA, and none of the animals got seroconverted [21]. Furthermore, intranasal inoculation of SARS-CoV-2 in pigs, chickens, and ducks could not have SARS-CoV-2 RNA in any of the rectal swabs collected from the virus-inoculated or naïve contact animals [34]. Thus, this study confirmed the non-susceptibility of pigs, chickens, and ducks to SARS-CoV-2.

In another study, five poultry species, including chickens (*Gallus gallus domesticus*), Japanese quail (*Coturnix japonica*), turkeys (*Meleagris gallopavo*), Pekin ducks (*Anas platyrhinchos domesticus*), and White Chinese geese (*Anser cygnoides*) were challenged with SARS-CoV-2 virus [49]. All these birds were inoculated with either the USA-WA1/2020 isolate of SARS-CoV-2 (BEI NR-58221) or the Florida/USA-2_SaudiArabia_2014 isolate of MERS-CoV (BEI NR-50415). Clinical signs, viral RNA, or viral-specific antibodies were not detected at any time in these birds, and the virus did not even replicate in embryonated chicken eggs (ECE). Thus, the study reported the unlikeliness of poultry playing a role in the maintenance or transmission of either SARS-CoV-2 or MERS-CoV and ECE being a non-viable laboratory host system.

## One Health Approach to Control the Reverse Zoonosis

COVID-19 pandemic has reminded us again about the importance of one health concept. One health approach is a potentially effective and coordinated collaboration of authorities of related fields to attain the most advantageous health outputs for the benefit of animals, humans, and the environment in affected regions and on a global level. In such an approach, the activities of professionals working in the human health, animal health, plant health, and the environment sectors are directed to follow integrated and systemized action plans which make sure the efficient sharing of relevant information and urgent strategy development in situations where delay in preventive measures could lead to irrepressible consequences. The implementation of the one health approach through the rapid coordination of the state, local, and federal system, public health, and animal health partners together reduced the potential risks associated with exposure to the SARS-CoV-2 virus in Chinese animal markets (believed to be the origin of COVID-19). In Canada, the development of machine learning algorithms by the Canadian Institutes of Health Research in collaboration with the Public Health Agency of Canada’s Internet-based Surveillance Informing Global Health Threats using the data collected from domestic and wild animals to assist the rapid detection of COVID-19 cases with real-time public health recommendations through electronic media.

Different reports across the globe show the spread of the virus from humans to animals during close contact. For the identification of SARS-CoV-2 infection in companion animals, the coordinated approach in drafting of policies and protocols, evaluating resources, and engaging partners at all levels have to be done by incorporating inputs from human and animal health experts. Thus early-stage prevention of disease spread both in human and in animal populations is possible, which, in turn, could stop the onset of an epidemic and thereby saving the health, environment and the economy. The SARS-CoV-2 infection in animals has to be addressed with extensive care and there should be systems to support the safe transportation of animals from the souse of infection (home, animal care centers, zoo, etc.) to the veterinary facilities. Strict disinfection procedures should be carried out to prevent the further spread to any other animals or reinfection possibilities. The guidelines have to be implemented regarding the “do’s and don’ts” for both the susceptible categories of humans and animals to safeguard against disease transmission. The local region-wise corporation of animal control, animal services, or an animal rescue group has to be coordinated. The animal handlers, whether in domestic or commercial environments, have to be trained for the implementation of biosafety procedures for infectious diseases such as social distancing, infection prevention measures, use of appropriate PPE, proper cleaning, and disinfection guidelines. There should be defined policies, equipment, and resources in place for the handling of test-positive animals in every animal organization. When an animal tests positive for SARS-CoV-2, immediate cohesive action is required between the public health veterinarians and animal health officials. If the animal is healthy or has mild symptoms, and can be exempted from hospitalization only on the strict adherence to infectious disease guidelines from the government, which include the daily monitoring, movement restrictions, isolation recommendations, safe handling and disposal of animal waste until the animal tests negative for the virus.

Another crucial strategy is to perform the combined epidemiological investigation by local, state, and federal one health partners to understand the circulation and spread of the virus under natural conditions. According to the Centers for Disease Control and Prevention (CDC), this investigation should include the following components: (a) details of the diagnostic and clinical factors used for the determination of the case, (b) risk factor assessment in the test-positive animal, including the potential source of infection, and (c) evaluation of potentially exposed people and animals [50]. The proper documentation of any positive cases of infection either in animals or humans should be done and their primary, secondary, and tertiary contacts have to be traced to control the risk of transmission and to have a good understanding of the SARS-CoV-2 zoonotic events. Moreover, the application of one health approach can reduce economic impacts on the livestock industry and food supply [51].

## Conclusion

The zoonotic and reverse zoonotic potentials of SARS-CoV-2 infection are identified in several animal species from different countries through serological, molecular analyses, and experimental infection studies. Literature suggests that SARS-CoV-2 can infect pets, other domestic and wild animals, making it a serious global issue. The cat, dog, mink, tiger, lion, snow leopard, and puma were confirmed to be susceptible to natural SARS-CoV-2 infection. Whereas, under laboratory conditions, experimental infection studies demonstrated that cats, ferrets, hamsters, Egyptian fruit bats, and non-human primates can be infected by SARS-CoV-2. These shreds of evidence suggest that pets/companion animals living in the household of people with COVID-19 are at risk of contracting the infection, which can further spread the virus to other naive animals. Some viral genomic studies also showed that SARS-CoV-2 in animals were identical to viruses detected in the people close to them, showing evidence for viral transmission between humans and animals. Thus the global COVID-19 pandemic, as well as later indications of the disease’s panzootic potential, underline the necessity for a One Health approach. Systematic guidelines for the monitoring and intervention in wild, captive, and companion animals must be set in place to have a better understanding of viral spread in host population. The emergence of the COVID-19 pandemic has challenged the world and emphasized the importance of the interconnectivity of global health-care systems and has led to considerable changes in societal behaviors and working practices. Effective collaboration in research and practice between medical and veterinary practitioners in partnership with biologists and environmentalists is required to alleviate the widespread global concern over environmental and public health issues.

## Authors’ Contribution

RKP: Selected the topic, conceptualized the review, conducted the literature review, extracted relevant information, and drafted the manuscript. AVN: Conducted literature review, extracted relevant information, and critically read the manuscript. PKT, KC, TVK, ARR, BMM, MA, and AKP: Critically read and commented on different versions of the manuscript. All authors read and approved the final manuscript.
